# Upregulated Talin1 synergistically boosts β-estradiol-induced proliferation and pro-angiogenesis of eutopic and ectopic endometrial stromal cells in adenomyosis

**DOI:** 10.1186/s12958-021-00756-7

**Published:** 2021-05-14

**Authors:** Yi-yi Wang, Hua Duan, Sha Wang, Yong-jun Quan, Jun-hua Huang, Zheng-chen Guo

**Affiliations:** 1 Department of Minimally Invasive Gynecologic Center, Beijing Obstetrics and Gynecology Hospital, Capital Medical University, No.17 Qi Helou Road, Dong Cheng District, Beijing, 100006 China; 2 Department of Urology, Beijing Tongren Hospital, Capital Medical University, Beijing, 100730 China

**Keywords:** Adenomyosis (ADS), β-Estradiol (β-E_2_), Talin1, Adenomyotic eutopic and ectopic endometrial stromal cell (ADS_Eu_ESC and ADS_Ec_ESC), Proliferation, Pro-angiogenesis

## Abstract

Adenomyosis (ADS) is an estrogen-dependent gynecological disease with unspecified etiopathogenesis. Local hyperestrogenism may serve a key role in contributing to the origin of ADS. Talin1 is mostly identified to be overexpressed and involved in the progression of numerous human carcinomas through mediating cell proliferation, adhesion and motility. Whether Talin1 exerts an oncogenic role in the pathogenesis of ADS and puts an extra impact on the efficacy of estrogen, no relevant data are available yet. Here we demonstrated that the adenomyotic eutopic and ectopic endometrial stromal cells (ADS_Eu_ESC and ADS_Ec_ESC) treated with β-estradiol (β-E_2_) presented stronger proliferative and pro-angiogenetic capacities, accompanied by increased expression of PCNA, Ki67, VEGFB and ANGPTL4 proteins. Meanwhile, these promoting effects were partially abrogated by Fulvestrant (ICI 182780, an estrogen-receptor antagonist). Aberrantly upregulation of Talin1 mRNA and protein level was observed in ADS endometrial specimens and stromal cells. Through performing functional experiments in vitro, we further determined that merely overexpression of Talin1 (OV-Talin1) also enhanced ADS stromal cell proliferation and pro-angiogenesis, while the most pronounced facilitating effects were found in the co-intervention group of OV-Talin1 plus β-E_2_ treatment. Results from the xenograft nude mice model showed that the hypodermic endometrial lesions from co-intervention group had the highest mean weight and volume, compared with that of individual OV-Talin1 or β-E_2_ treatment. The expression levels of PCNA, Ki67, VEGFB and ANGPTL4 in the lesions were correspondingly elevated the most in the co-intervention group. Our findings unveiled that overexpressed Talin1 might cooperate withβ-E_2_ in stimulating ADS endometrial stromal cell proliferation and neovascularization, synergistically promoting the growth and survival of ectopic lesions. These results may be beneficial to provide a new insight for clarifying the pathogenesis of ADS.

**Supplementary Information:**

The online version contains supplementary material available at 10.1186/s12958-021-00756-7.

## Introduction

Adenomyosis (ADS) is a commonly encountered benign gynecological disorder, predominantly occurring in women of reproductive age. It is typically characterized as the aberrant displacement of eutopic uterine endometrial glands and stroma, deeply and haphazardly involved into myometrium [1]. The concomitant myometrial hyperplasia and hypertrophy may be associated with a series of subinfertility. However, the only radical treatment strategy is still hysterectomy, by which fertility preservation is compromised. In particular, the pathogenesis of ADS remains uncertain as yet, therefore, individualized therapy and targeted agents are still difficult to achieve.

According to the invagination and EMID (endometrial-myometrial interface disruption) theory [2, 3], ADS may actually derives from the excessive invasion of altered endometrial basalis into myometrium, after passing through the disrupted EMI. Despite the mechanism that triggers the progressive invasion of endometrium has not been fully elucidated, accumulating evidence supported that there were biochemical and functional abnormalities related to the endometrial cells. It has been postulated that sustained proliferation and survival of eutopic or ectopic endometrial cells may, along with enhanced migratory prosperities, permit the deeper invasion and down-growth of ectopic lesions [4, 5]. Meanwhile, angiogenesis is considered to be an essential component during the development of ADS, as the implantation of ectopic endometrium requires a blood supply to maintain its growth and survival [6].

ADS is a recognized estrogen-dependent disease. Thus far, multiple observations have implied that elevated local E_2_ concentration and ER overexpression might be central and crucial to the pathogenesis of ADS. Based on the available data, the local hyperestrogen milieu may result in increased endometrial cell proliferation, enhanced pro-angiogenetic prosperity and induction of epithelial-mesenchymal transition (EMT), thereby promoting the implantation and invasion of ectopic endometrium into myometrium, eventually contributing to the onset of ADS [4, 7, 8]. However, whether the effects of active estradiol (β-E_2_) on adenomyotic endometrium could be altered by certain pathogenic genes, the direct evidence is still limited.

Talin1, a ubiquitous intracellular cytoskeletal protein containing 2541 amino acids, is a key regulator of integrin activation [9]. It has been well identified that Talin1 is closely connected with the progression of multiple human cancers through mediating cell proliferation, migration and invasion [10]. Previously, we have demonstrated that aberrantly overexpressed Talin1 might induce EMT to facilitate ADS endometrial epithelial cell migration and invasion via activating wnt/β-catenin pathway [11]. However, whether Talin1 serves an extra role in β-E_2_-induced proliferation and angiogenesis of adenomyotic endometrium, and then affects the invasive implantation of ectopic lesions, it remains undiscovered yet.

Therefore, the main purpose of the study was to investigate whether Talin1 participates in the development of ADS by directly influencing the regulatory effects of β-E_2_ on proliferation and pro-angiogenesis of endometrium in vitro and in vivo.

## Materials and methods

### Sample collection

All tissue samples were collected with informed consent. Our study was in accordance with the requirements of the Medical Ethics Committee of Beijing Obstetrics and Gynecology Hospital, Capital Medical University (IEC-C-29-V02-FJ1). The eutopic and corresponding ectopic uterine endometrium were obtained during hysterectomy from 28 women diagnosed with ADS, which were utilized for subsequent primary culture of endometrial stromal cells. Meanwhile, normal endometrial tissues were collected from 22 women undergoing hysterectomies for benign ovarian tumors or cervical intraepithelial neoplasia II-III, without histological evidence of ADS. All participants were premenopausal with regular menstrual cycles and at proliferative phase during the procedure. Any signs or symptoms of pathologic changes in endometrium, a history of hormone therapy within 3 months, or concomitant with endometriosis were not included in the study [12]. All endometrial specimens were obtained from February 2019 to January 2020.

### Cell culture

As we reported previously [13], the primary adenomyotic eutopic and ectopic endometrial stromal cells (ADS_Eu_ESC and ADS_Ec_ESC, *n* = 7 respectively) as well as the normal uterine endometrial stromal cells as control (Ctrl_ESC, *n* = 5) were isolated from the corresponding endometrium and cultured in vitro. Briefly, after being rinsed with PBS 2 ~ 3 times to remove impurities and blood cells, the separated endometrial specimen was then minced into pieces less than 2mm^3^. Subsequently, 0.02% type I collagenase (Sigma, USA) mixed with 0.005% deoxyribonuclease (Invitrogen, USA) was added to aptly digest the tissue debris for 45 ~ 60 min at 37 °C. To accelerate the digestion process, a gentle shaking every 5 ~ 10 min was helpful. Afterwards, DMEM/f12 (Hyclone, USA) containing 12.5% fetal bovine serum (FBS, BD, USA) was utilized for stopping the digestion. Filtered through the 100 μm cell strainer, the cell suspension free from mucosa and remnants was obtained. After successive centrifugation at room temperature (720 rpm, 3 min) and filtration through the 40 μm cell strainer, the filtrate was then centrifuged twice (1200 rpm, 3 min). Finally, the primary uterine endometrial stromal cells were isolated and seeded in culture dishes. Cells were cultured with DMEM/f12 medium containing 12.5% FBS and 1% penicillin/streptomycin (Gibco, USA). When reaching 80% confluence, the primary cells were trypsinzed and passaged. We selected the cells at (Passage 3 to Passage 6) P_3_-P_6_ for subsequent experiments. (Supplementary Figure S1).

The estrogen receptor positive (ER^+^) Ishikawa cells (Human Asia endometrial adenocarcinoma cell line) and HUVECs (human umbilical vein endometrial cells) were purchased from China Infrastructure of Cell Line Resource and cultivated in DMEM/f12 medium with 10% FBS. All cells were incubated at 37 °C and 5% CO_2_ in a humidified atmosphere.

### Drug treatment

To remove the confounding effects of endogenous steroids, ADS_Eu_ESC, ADS_Ec_ESC and Ishikawa^ER+^ cells were cultured in phenol red-free DMEM/f12 medium for 48 h before drug treatment. Subsequently, cells were incubated in fresh medium (as control), β-E_2_ (10nM, Sigma, USA), Fulvestrant (10 nM, ICI 182780, a selective ER antagonist, MedChem Express, USA) or β-E_2_ plus Fulvestrant for 24 h.

### Gene regulation

The lentivirus vectors containing Talin1 overexpression plasmid pSGLV (OV-Talin1) and its corresponding negative control (OV-NC) were constructed by Gene Chem (Shanghai, China). Meanwhile, the short hairpin RNA (ShRNA) against Talin1 (Sh-Talin1) and its negative control ShRNA (Sh-NC) were also synthesized and generated by Gene Chem. The ADS_Eu_ESC, ADS_Ec_ESC and Ishikawa^ER+^ cells were firstly seeded into 6-well plates at a density of 2х10^5^ cells/well. When reaching 50 ~ 60% cell confluence, the OV-Talin1 or OV-NC vector was transfected into cells using a lipofectime 3000 (Invitrogen, USA) according to the manufacturer’s instructions. Meanwhile, the cells were subjected to lentivirual transduction with 5 μg/ml polybrene for 24 h, and the medium was then changed. A qRT-PCR or western blot assay was performed for further detecting the transfection efficiency. Especially, the ADS_Eu_ESC and ADS_Ec_ESC cells transfected with OV-Talin1 continued to receive 10 nM β-E_2_ treatment for 24 h, as mentioned above, after removal of the endogenous steroid hormones in the phenol red-free medium.

### Quantitative reverse transcription-polymerase chain reaction (qRT-PCR)

The RNA isoPlus (Takara, BioInc, Japan) was used for the total RNA extraction from endometrial tissue samples and cells. For the reverse transcription of Talin1, the PrimeScript RT Reagent Kit (Takara) was utilized to synthesize the cDNA. The subsequent quantitative PCR traction was performed following the protocol of a SYBR Green PCR Kit (Takara) through an ABI 7500 system (Applied Biosystems, Grand Island, USA). The thermocycling conditions were as follows: 95 °C for 5 s and 60 °C for 30 s. β-actin was selected as the reference gene. The experiment was independently repeated for 3 times and the results were analyzed with 2^-ΔΔCT^ method. The following primers specific to Talin1 were used for quantitative real-time PCR: sense primer, 5′-CTATATGCCACACCCGCCTC-3′ and antisense primer, 5′-CCCAGGATTCCACGGGACTA-3′. The primers for internal control β-actin were as follows: forward, 5′-GCCGTGGTGGTGAAGCTGT-3′ and reverse, 5′-ACCCACACTGTGCCCATCTA-3′. All the primers in the study were generated by Sangon Biotech (Shanghai, China).

### Western blot

As previously described [14], total protein from each sample was extracted with RIPA lysis buffer (Sigma, St, Lousis) containing 1 mmol/L phenylmethylsulfonyl fluoride (PMSF, Solarbio). A phosphorylase inhibitor and a protease inhibitor cocktail (1:100, Solarbio) were added to prevent degrading of proteins in the extracts. tThe protein concentration was quantified using a BCA Protein Assay Kit (Beyotime, China). Afterwards, the equal boiled protein samples (30 μg) were loaded on 8 ~ 12% sodium dodecyl sulfatepolyacrylamide gels (Solarbio Life Sciences), electrophoretically separated, and transferred to a polyvinylidene fluoride (PVDF) membrane (Millipore, Billerica, Massachusetts), which had been wetted in 100% methanol for 15 s previously.. Then the membranes were blocked in 5% skim milk containing Tris-buffered Saline Tween (TBST, Solarbio, China) at room temperature for 1 h to reduce nonspecific bindings. Subsequently, the membranes were incubated with the primary antibodies (1:500 ~ 1:1000 dilutions, Cell Signaling Technology, USA) overnight at 4 °C with gentle agitation. Getting washed 2 ~ 3 times with TBST, the secondary antibodies (1:2000 dilution) were added for incubation for 1 h. Finally, the immunoreactive bands were detected with Chemiluminescent HRP Substrate (Merck Millipore) and quantified through a Image Lab software (Bio-Rad, Hercules, USA) .

### Cell viability assay

After receiving the drug treatment and/or gene regulation as described above, the ADS_Eu_ESC and ADS_Ec_ESC cells were seeded in 96-well plates (4 × 10^3^ cells /well) and cultured for 96 h. Then 10 μl/well cell-counting kit-8 (CCK-8, Dojindo, Japan) reagent was added at indicated time points (24 h, 48 h, 72 h, 96 h) and the corresponding cells were incubated for another 1 h at 37 °C. The absorbance of each well at 450 nm was measured with a microplate reader (Bio-Rad, USA) and cell viability was evaluated.

### Plate colony formation assay

The ADS_Eu_ESC and ADS_Ec_ESC cells were seeded in 6-well plates at a density of 2 × 10^3^ cells /well. Thereafter, cells were incubated for 14 days to allow colony formation, during which the medium was refreshed every 2 ~ 3 days. Then 1 ml/well 4% paraformaldehyde and 0.1% crystal violet were used to fix and stain the cells, respectively. Eventually, the number of visible colonies was counted after full decolorization.

### Capillary tube and network formation assay

The pro-angiogenetic activity of ADS_Eu_ESC and ADS_Ec_ESC was evaluated by a capillary tube and network formation assay. Firstly, 24-well ice-cold plates were coated with 250 μl/well Matrigel (BD, Bioscience, USA, 1:4 dilution in serum-free DMEM/f12) and incubated for 30 min at 37 °C. Meanwhile, the ADS_Eu_ESC and ADS_Ec_ESC cells with different treatment were collected, centrifuged and filtered to obtain the corresponding conditioned medium. Then HUVECs (2 × 10^3^, P_2_-P_4)_ in 250 μl conditioned medium were overlaid on the Matrigel. After incubation at 37 °C for 6 h, 4 μM Calcein Acetoxymethyl Ester (Calcein AM) was added for cell staining followed by incubation for 30 min. Finally, after replacement with fresh medium, the number of new capillary formation was observed and counted under a fluorescence microscope.

### Xengraft model establishment and treatment

A total of 33 BALB/c female nude mice (4 week old) were purchased from Beijing Vital River Laboratory Animal Technology. Co., Ltd. (Beijing, China). The guidelines for animal care were approved by the committee on Animal Study of Beijing Obstetrics and Gynecology Hospital, Capital Medical University. The mice were raised under specific pathogen-free (SPF) conditions. All of them got bilaterally ovariectomized and left untreated for 2 weeks.

The Ishikawa^ER+^ cells transfected with OV-Talin1, OV-NC, treated with β-E_2_ or β-E_2_ + OV-Talin1 were made into single cell suspension (2 × 10^7^/ml) with a mixture of 200 μl PBS and 50 μl Matrigel. Subsequently, the cell suspension was delivered by subcutaneous injection into the right axilla lesions of nude mice. The length (L), width (W) and height (H) of the nodule lesions were measured every week and the lesion volumes were calculated according to the following formula [15]: V = π/6 (L x W x H). On the 84th day after cell inoculation, the mice were euthanized. Then the exfoliated lesion nodules were weighted and stored for subsequent experiment.

### Statistical analysis

Experiments were performed triplicately or more for statistical significance. The results were analyzed using SPSS 23.0 and Graphpad Prism software. The measurement data were expressed as mean ± standard deviation. Continuous variables in two groups were analyzed by independent sample t-test. One-way analysis of variance (ANOVA) was used for comparison of multiple groups. The difference was considered statistically significant at *P* < 0.05.

## Results

### β-E_2_ induced adenomyotic endometrial stromal cell overproliferation in vitro

**Fig. 1:**
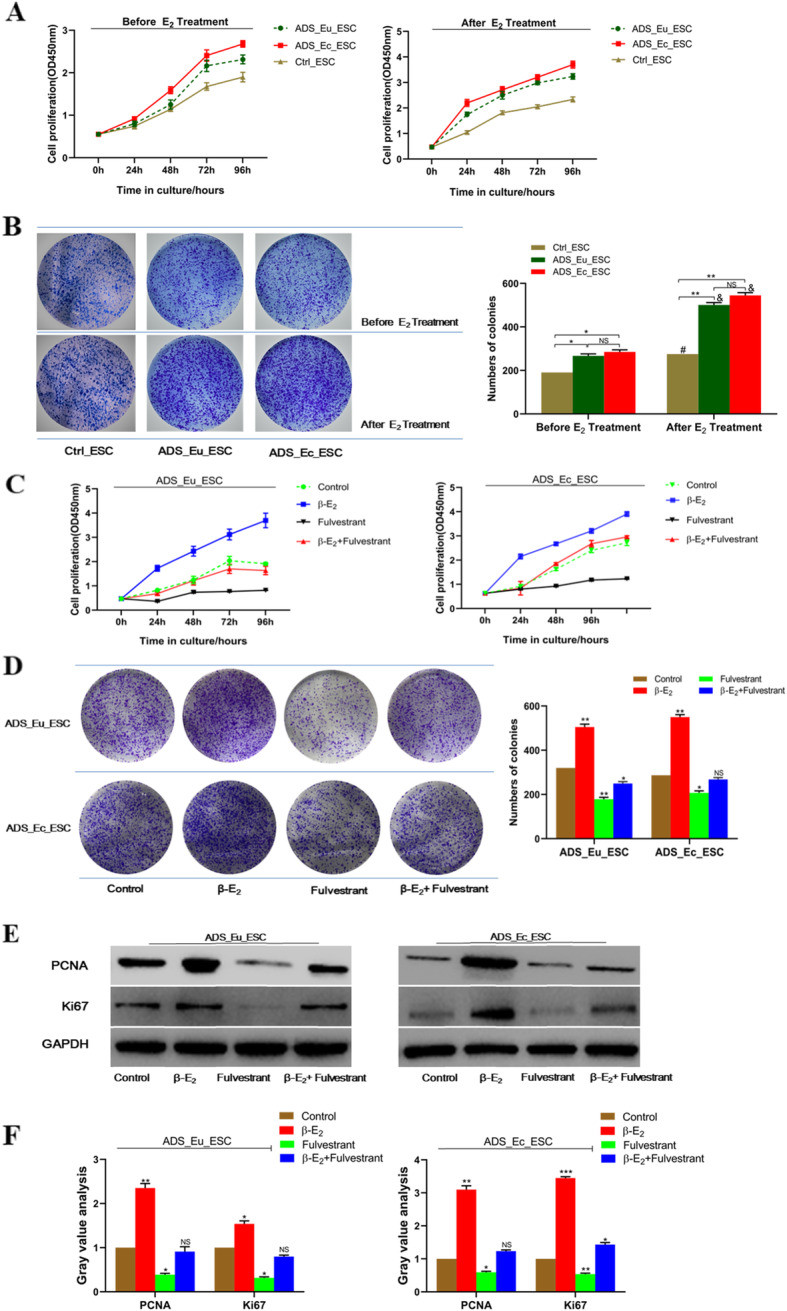
β-E_2_ induced adenomyotic endometrial stromal cell proliferation in vitro. **a**. Cell proliferation rates of adenomyotic and normal endometrial stromal cells with or without **β-**E_2_. **b**. Numbers of formative colonies of adenomyotic and normal endometrial stromal cells with or without **β-**E_2_. **c**. and **d**. CCK-8 assay and colony formation assay were performed to evaluate the effects of **β-**E_2_ on the proliferation of ADS eutopic and ectopic endometrial stromal cells. **e**. and **f**. Western blot and gray value analysis of molecular markers for cell division and proliferation including PCNA and Ki67 in ADS endometrial stromal cells treated with E_2_ (10nM) or Fulvestrant (a selective estrogen receptor antagonist, 10 nM) or both or neither of them. GAPDH was used as a loading control. Data were presented as mean ± SD. ^NS^ no significance, ^*^P < 0.05, ^**^P < 0.01. ADS_Eu_ESC, adenomyotic eutopic endometrial stromal cells; ADS_Ec_ESC, adenomyotic ectopic endometrial stromal cells

In view of previous studies, it has been reported that a local hyperestrogenic milieu might serve a key role in the progression of ADS. Therefore, we further investigated how β-E_2_ affected endometrial stromal growth and proliferation. As presented in Fig. 1a and b, cell proliferation rates of ADS_Eu_ESC and ADS_Ec_ESC were both significantly higher than that of Ctrl_ESC cells regardless of β-E_2_ intervention, while there was no statistical difference between the two ADS endometrial cells. After a 10 nM-E_2_ stimulation, the proliferation rates of the two ADS cells increased notably compared with that of Ctrl_ESC cells. In addition, compared with Ctrl_ESC cells, ADS endometrial cells still had more clone formative numbers whether treated with E_2_ or not. Especially, the two ADS cells had more obvious responses toβ-E_2_-induced colony formation.

On the basis of clarifying the different effects of β-E_2_ on ADS and normal endometrial cell proliferation, we further demonstrated that a suitable dose of 10 nM β-E_2_ treatment provided the most significant promoting effects on proliferative rates and colony formative ability, no matter for ADS_Eu_EEC or ADS_Ec_ESC cells (Fig. 1c, d). Meanwhile, the 10 nM of Fulvestrant (ICI 182780, a selective ER antagonist) notably abrogated β-E_2_-induced cell proliferation in vitro. Furthermore, PCNA (Proliferating Cell Nuclear Antigen) and Ki67 were detected through western blot assay as the molecular markers for cell division and proliferation. As expected, treatment with β-E_2_ presented a stronger activating effect on the expression of PCNA and Ki67 in the two ADS endometrial stromal cells, whereas the Fulvestrant alone inhibited the two protein levels. Consistent with the functional experiments above, the co-treatment with β-E_2_ and Fulvestrant also partially abolished the overexpression of PCNA and Ki67 induced by β-E_2_ (Fig. 1e, f). These findings indicated that β-E_2_ could facilitate ADS endometrial stromal cell proliferation in an ER-dependent manner.

### β-E_2_ induced adenomyotic endometrial stromal cell pro-angiogenesis in vitro

**Fig. 2:**
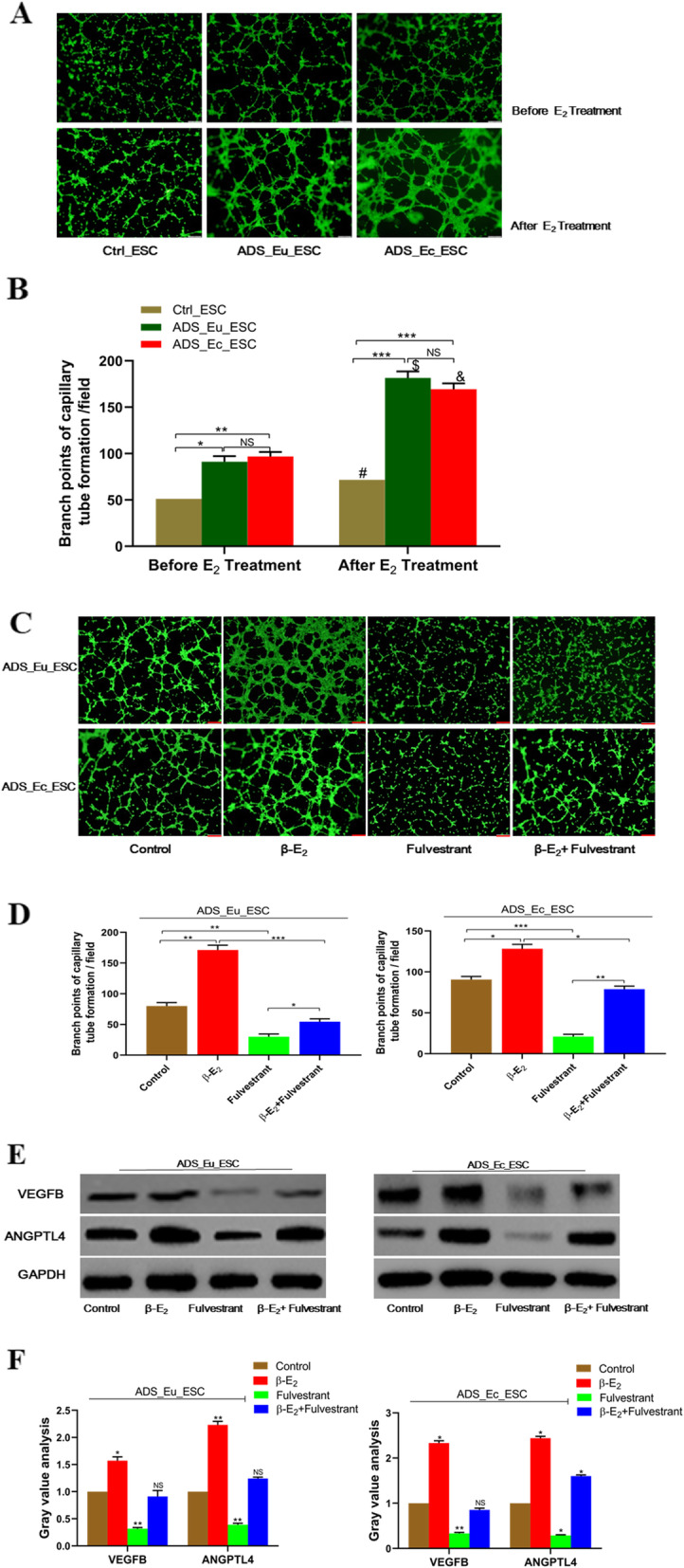
β-E_2_ induced adenomyotic endometrial stromal cell angiogenesis in vitro. **a**. **b**. Assessment of pro-angiogenetic ability of adenomyotic and normal endometrial stromal cells with or without **β-**E_2_. **c**. **d**. Tube and network formation assay of HUVEC cells treated with conditioned media (the whole cell filtrate from ADS_Eu_ESC and ADS_Ec_ESC added with either E_2_ (10nM) or Fulvestrant (10 nM) or both. Images were captured when HUVEC were cultured with the conditioned media on Matrigel for 6 h. Branch points of capillaries were counted and analyzed. Scale bar = 100 μm. **e**. **f**. Western blot detection of angiogenesis-related proteins including VEGFB and ANGPTL4 in ADS_Eu_ESC and ADS_Ec_ESC cells after being treated with E_2_ (10nM) or Fulvestrant (10 nM). GAPDH was used as a loading control. Data were presented as mean ± SD. ^*^P < 0.05, ^**^P < 0.01, ^***^P < 0.001. HUVEC, human umbilical vein endothelial cells

Since human umbilical vein cells (HUVECs) has been identified to be a well-established model for angiogenesis, we further conducted the capillary tube formation assay to investigate the impacts of β-E_2_ on the pro-angiogenetic potential in HUVECs. As Fig. 2a and b illustrated, no matter whether β-E_2_ was given or not, the pro-angiogenetic capacity of ADS_Eu_ESC and ADS_Ec_ESC cells was higher than that of Ctrl_ESC, despite no statistical difference was observed between the two ADS cells. Moreover, when cells were treated with 10 nM β-E_2_ for 6 h, compared with Ctrl_ESC cell, the numbers of neovascular branch points in HUVECs were both enhanced more by the two ADS cells, indicating the varying degrees of effects of estrogen on ADS and normal endometrial cell proangiogenesis. Furthermore, the conditioned medium from ADS_Eu_ESC treated with 10 nM of β-E_2_ was able to induce the vascular endothelial cell capillary tube and network formation, while this promoting effect was reversed by additional treatment of Fulvestrant. Similarly, the branch points of neovascularization were also the most in HUVECs cultivated with medium from ADS_Ec_ESC receiving β-E_2_ activation. Meanwhile, the Fulvestrant blocked the pro-angiogenetic capacity of ADS_Ec_ESC (Fig. 2c and d). Correspondingly, we also demonstrated that a dose of 10 nM β-E_2_ had a pronounced induction on the overexpression of VEGFB (vascular endothelial growth factor B) and ANGPTL4 (Angiopoietin-like Protein 4), the two representative proangiogenetic factors, in ADS_Eu_ESC cells. In contrast, both VEGF and ANGPTL4 showed the lowest expression levels in ADS_Eu_ESC cells treated with the Fulvestrant. The similar tendency was also observed in ADS_Ec_ESC (Fig. 2e, f). Taken together, a suitable dose of β-E_2_ could enhance the pro-angiogenetic activity of ADS endometrial stromal cells and the effect got suppressed by the ER antagonist treatment.

### Talin1 was upregulated in ADS endometrial tissues and cells

**Fig. 3:**
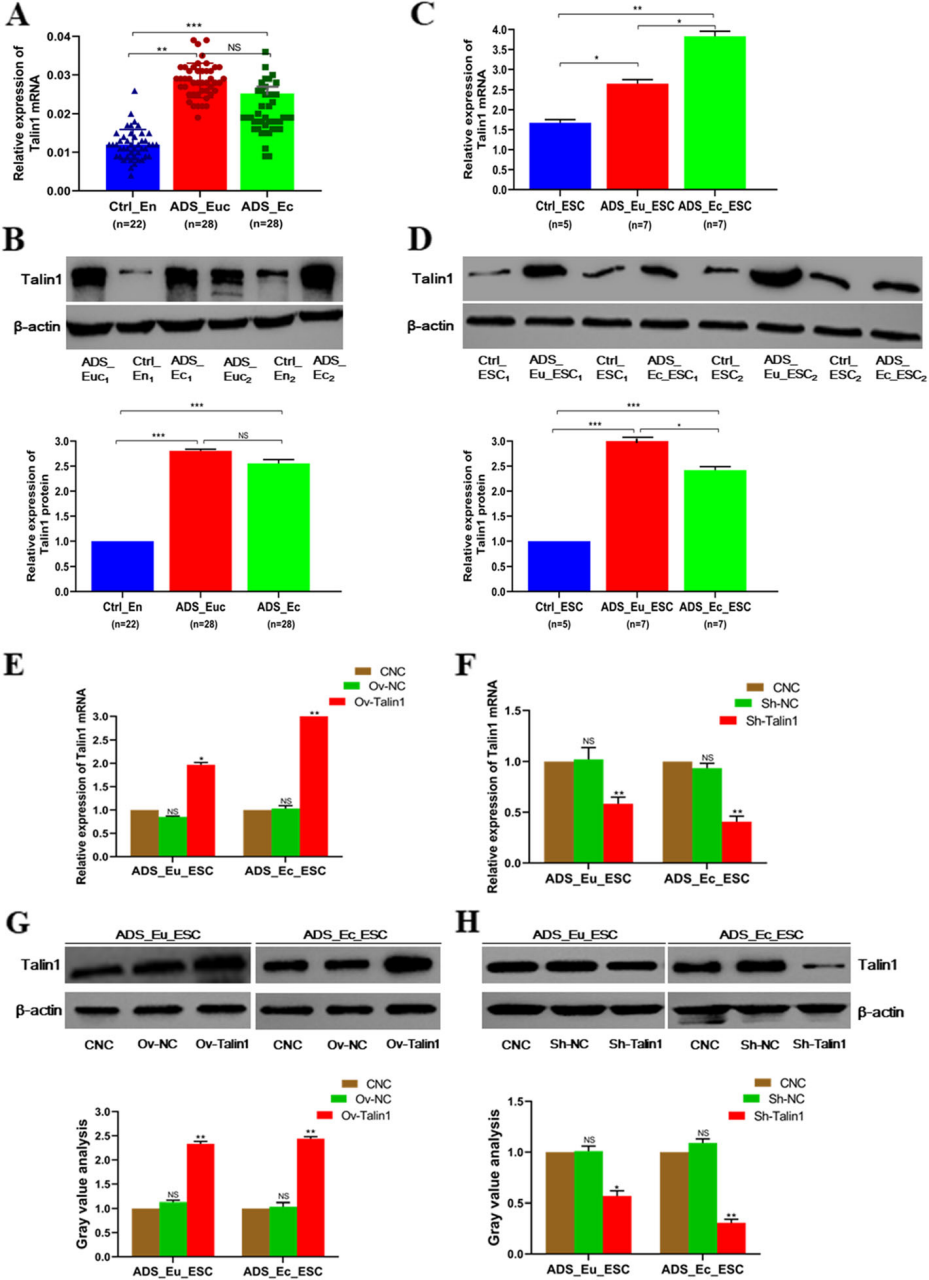
Talin1 was overexpressed in human ADS uterine endometrial tissue and cells. **a**. The relative expression of Talin1 mRNA was elevated in ADS_Euc (*n* = 28) and ADS_Ec (n = 28) endometrium, as detected by qRT-PCR and normalized against Ctrl_En (*n* = 22) endometrium. **b**. Western blot analysis for Talin1 protein relative expression levels in ADS eutopic and ectopic endometrium as well as the normal uterine endometrium. **c**. qRT-PCR analysis of Talin1 mRNA in ADS and normal uterine endometrial stromal cells. **d**. Western blot detection and gray value analysis of Talin1 protein in ADS and normal uterine endometrial stromal cells. β-actin was used as a loading control. **e-f**. Evaluation on the upregulated efficiency of Talin1 in ADS endometrial stromal cells. **g-h**. Evaluation on the knockdown of Talin1 in ADS endometrial stromal cells. Data were presented as mean ± SD. ^NS^ no significance, ^*^P < 0.05, ^**^P < 0.01, ^***^P < 0.001. Ctrl_En, normal uterine endometrium as control; ADS_Euc, eutopic endometrium of adenomyosis; ADS_Ec, ectopic endometrium of adenomyosis; Ctrl_ESC, normal uterine endometrial stromal cells as control; ADS_Eu_ESC, adenomyotic eutopic endometrial stromal cells; ADS_Ec_ESC, adenomyotic ectopic endometrial stromal cells. CNC, complete negative control (cells without any treatment); Ov-NC and Sh-NC, overexpression and knockdown vector for negative control, respectively; Ov-Talin1 and Sh-Talin1, overexpression and knockdown of Talin1, respectively

Consistent with the results from our previous studies, the basal expression level for Talin1 mRNA was significantly higher both in the eutopic and ectopic endometrium of ADS (ADS_Euc and ADS_Ec group, respectively) than that of control group (Ctrl_En), while no statistical difference was observed between the ADS_Euc and ADS_Ec group (Fig. 3a). Correspondingly, as Fig. 3b illustrated, the Talin1 protein was notably overexpressed in the two ADS endometrium. Despite there was a slight increase in ADS_Euc than that of ADS_Ec, the difference of Talin1 protein level between the two groups presented no statistical significance. As expected, we demonstrated the relative expression of Talin1 mRNA in ADS ectopic endometrial stromal cells (ADS_Ec_ESC) exerted higher than that of control (Ctrl_ESC) and ADS eutopic endometrial stromal cells (ADS_Eu_ESC) (Fig. 3c). Interestingly, an inspiration of data in Fig. 3d revealed that Talin1 protein level in ADS_Eu_ESC was the highest among the 3 endometrial stromal cells.

In the light of overexpression of Talin1 in ADS endometrial tissue and stromal cells, we supposed that Talin1 might play an oncogenic role in the onset and development of ADS. A Talin1 overexpression vector (Ov-Talin1) was transfected into ADS_Eu_ESC and ADS_Ec_ESC to upregulate its specific expression. Simultaneously, in order to take an inclusive view of the biological role that Talin1 played, a lentivirus vector Sh-Talin1 was also constructed and transfected into the above two ADS endometrial stromal cells to suppress Talin1 expression. The interference efficiency got verified by qRT-PCR and western blot assay (Fig. 3e-h).

### Knockdown of Talin1 repressed ADS endometrial stromal cell proliferative and pro-angiogenetic capacity

**Fig. 4:**
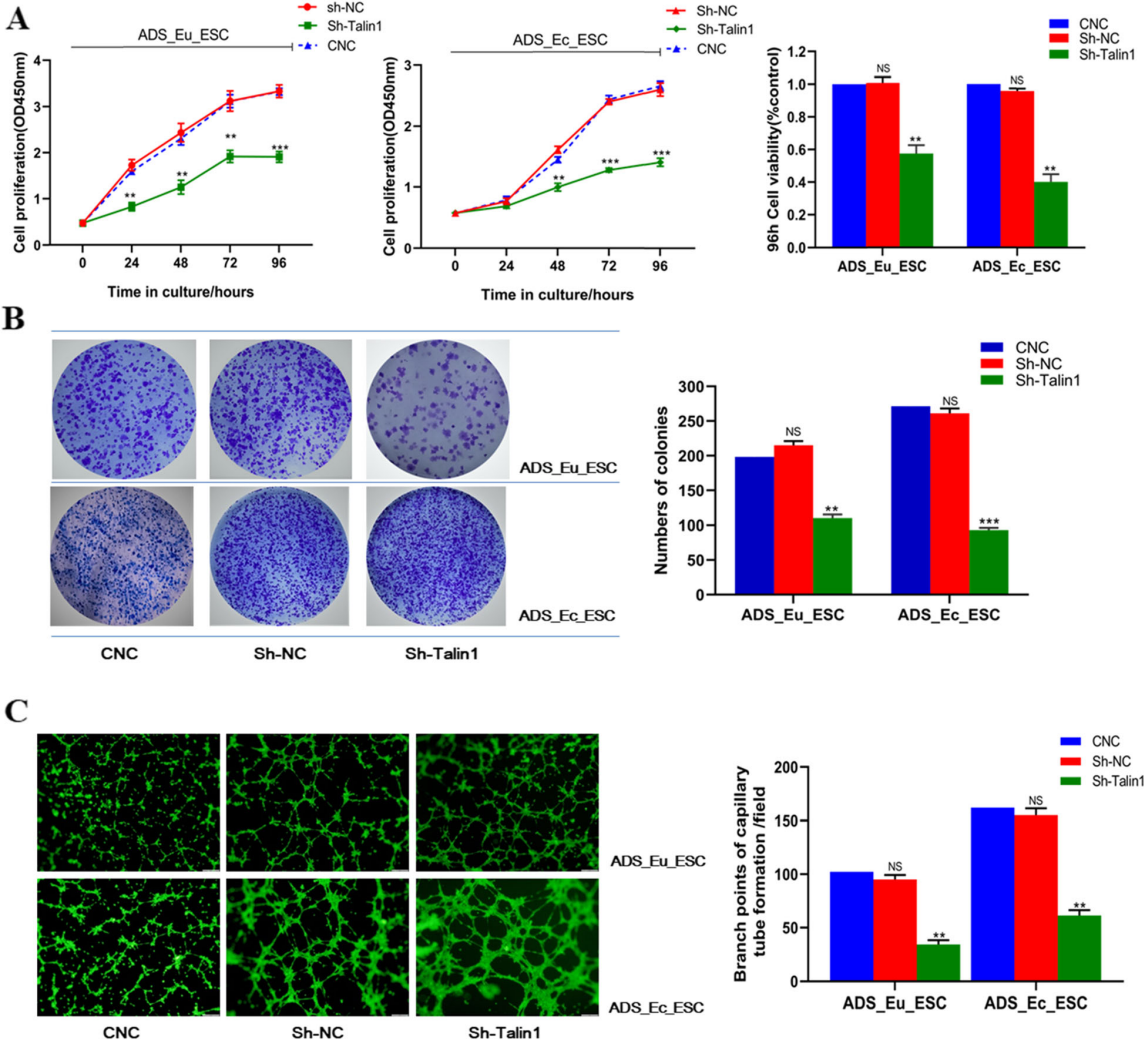
Knockdown of Talin1 repressed ADS endometrial stromal cell proliferative and pro-angiogenetic capacity. **a**. Cell Counting Kit-8 (CCK-8) assay was performed to assess the effect of Sh-Talin1 on the adenomyotic eutopic and ectopic endometrial stromal cell proliferation. The histogram illustrated the cell viability of ADS_Eu_ESC and ADS_Ec_ESC determined by the CCK-8 assay at 96 h post-transfection. **b**. Effect of Talin1 knockdown on the colony formation ability of ADS_Eu_ESC and ADS_Ec_ESC. **c**. Evaluation on the pro-angiogenetic ability of ADS_Eu_ESC and ADS_Ec_ESC cells transfected with Sh-Talin1 through the capillary tube and network formation assay. Images were captured when HUVECs were cultured with the conditioned medium on Matrigel for 6 h. Branch points of capillaries were counted and analyzed. Scale bar = 100 μm. At least triplicate independent experiments were performed. All data were presented as mean ± SD. ^*^P < 0.05, ^**^P < 0.01, ^***^P < 0.001. HUVEC, human umbilical vein endothelial cells

In view of the overexpression of Talin1 in ADS endometrial tissues and stromal cells, we speculated that Talin1 might serve an oncogenic or promoting role in the progress of the disease. To further investigate the biological effects of Talin1, we transfected the two ADS endometrial stromal cells with Sh-Talin1 to downregulate its expression specifically. The interference efficiency has been verified successfully, as we have mentioned above. As illustrated in Fig. 4b and Fig. 4c, both ADS_Eu_ESC and ADS_Ec_ESC cell growth got markedly inhibited once Talin1 was downregulated (Sh-Talin1), represented by the decreased proliferation rates and less colony formation in vitro. Meanwhile, the results from capillary tube and network formation assay demonstrated that the pro-angiogenetic ability of ADS_Eu_ESC and ADS_Ec_ESC cells was notably weakened in the Sh-Talin1 group (Fig. 4c). Consequently, these results implied that aberrantly overexpressed Talin1 might facilitate ADS endometrial stromal cell proliferation and subsequent survival in the ectopic myometrium, which might share some positive or simultative impacts during the β-E_2_-induced pathophysiology of this disease.

### Talin1 cooperated with β-E_2_ in facilitating adenomyotic endometrial stromal cell proliferation and pro-angiogenesis

**Fig. 5:**
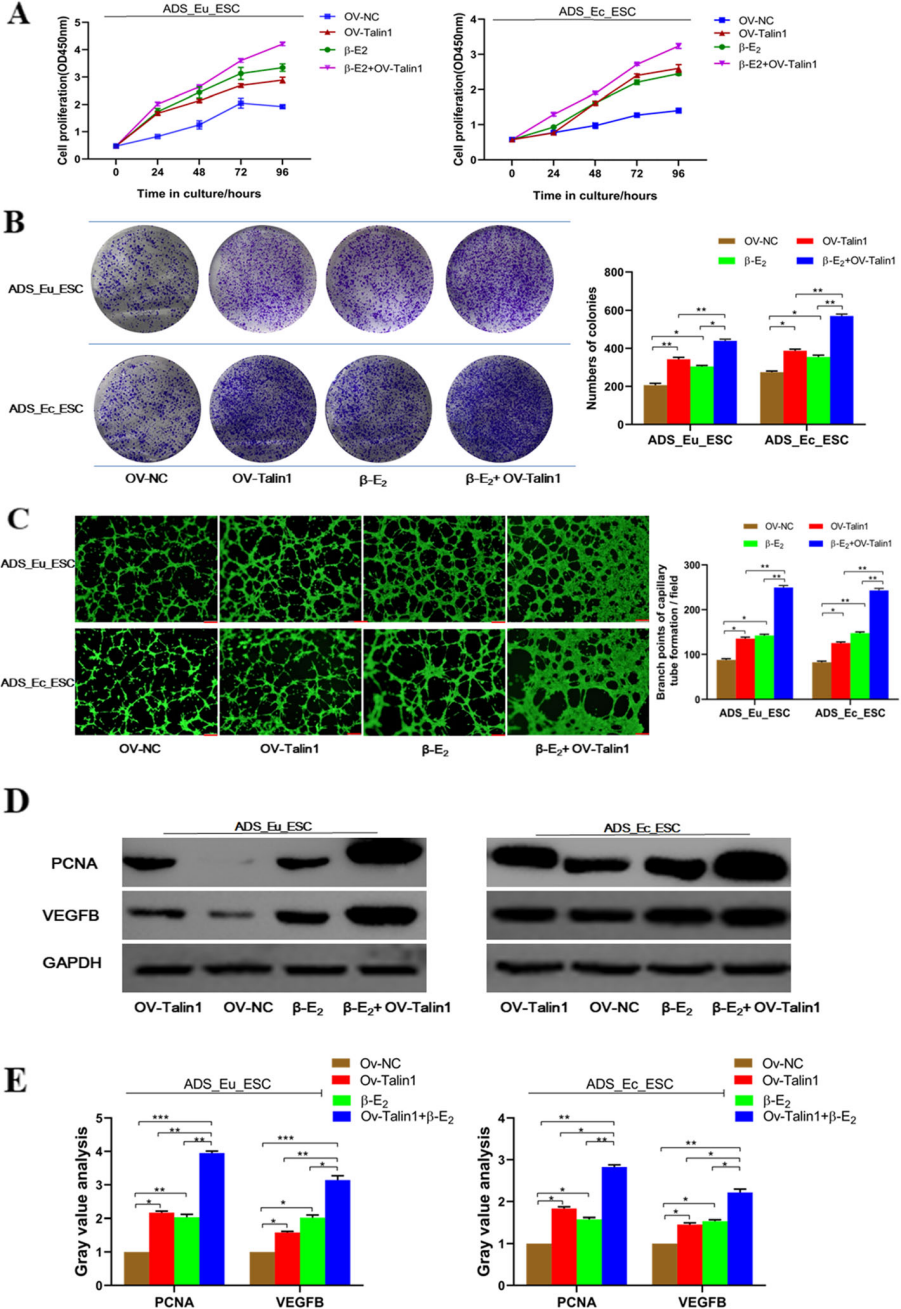
Talin1 cooperated with β-E_2_ in facilitating adenomyotic endometrial stromal cell proliferation and angiogenesis **a**. and **b**. Overexpression of Talin1 or treatment with **β-**E_2_ both promoted cell proliferation of ADS_Eu_ESC and ADS_Ec_ESC; while co-transfection of OV-Talin1 and **β-**E_2_ treatment showed the most significant enhanced effect on cell proliferation. **c**. Tube and network formation assay of HUVEC was performed to examine the angiogenetic ability of ADS_Eu_ESC and ADS_Ec_ESC treated with either OV-Talin1 transfection or **β-**E_2_ or both. **d**.**e**. Western blot detection of proteins related to cell proliferation and angiogenesis in adenomyotic endometrial stromal cells. GAPDH was used as a loading control. Data were presented as mean ± SD. ^*^P < 0.05, ^**^P < 0.01. OV-NC, negative control for Talin1transfection; OV-Talin1, overexpression of Talin1; **β-**E_2_ + OV-Talin1, after being transfected with Talin1 overexpression vector, cells were then treated with **β-**E_2_ (10nM)

Based on validating that the aberrant overexpressed Talin1 could enhance cell proliferative and pro-angiogenetic capacity in ADS, together with the promoting effects of β-E_2_ on ADS stromal cell proliferation and neovascularization, we further explored whether Talin1 and β-E_2_ could serve a synergistic role in the disease process. As displayed in Fig. 5a and Fig. 5b, the speed of cell proliferation and numbers of formative colonies both got increased in ADS_Eu_ESC treated with OV-Talin1 vector or 10 nM β-E_2_. Meanwhile, co-treatment of OV-Talin1 transfection and β-E_2_ addition showed the most significant enhanced effect on ADS_Eu_ESC cell proliferation. The similar results were observed in ADS_Ec_ESC cells. Results from the capillary tube formation assay suggested that no matter whether OV-Talin1 was transfected or β-E_2_ was treated alone, the conditioned medium from the two ADS cells could promote the neovascular sprouting in HUVECs, whereas the most pronounced angiogenesis-facilitating effect was found in the co-intervention group of β-E_2_ plus OV-Talin1 (Fig. 5c). In addition to cellular functional experiments, results from western blot assay simultaneously indicated that the proteins related to cell proliferation (PCNA) and angiogenesis (VEGFB) were the most upregulated in ADS_Eu_ESC and ADS_Ec_ESC co-treated with β-E_2_ plus OV-Talin1, although OV-Talin1 or β-E_2_ alone increased the proteins expression in contrast to the untreated cells (Fig. 5d,e). Given these findings, abnormally overexpression of Talin1 might cooperate with β-E_2_ in raising the proliferation and pro-angiogenesis of endometrial stromal cells, thus collectively stimulating the onset and progress of ADS.

### Additive effects of Talin1 on β-E_2_ stimulative growth and neovascularization of the hypodermic endometrial lesions in nude mice

**Fig. 6:**
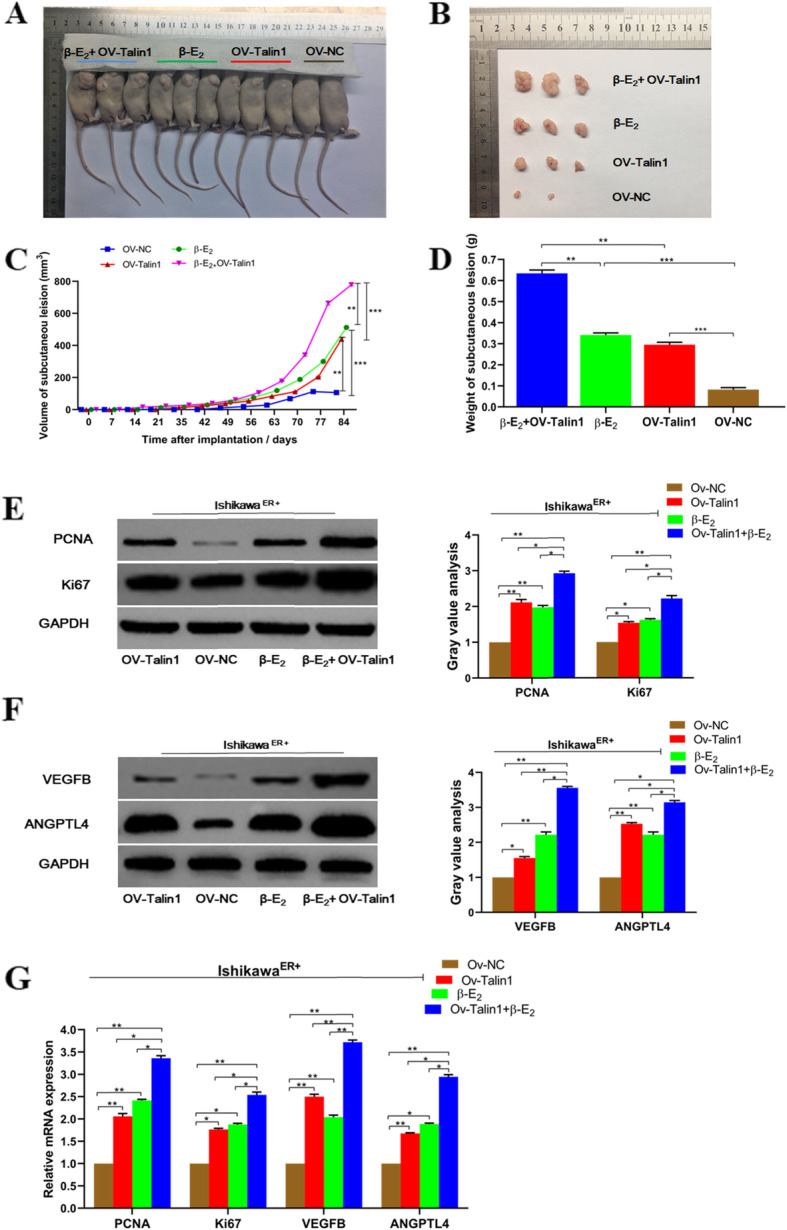
Additive effects of Talin1 on β-E_2_ promoted the growth and angiogenesis of hypodermic endometrial lesions in nude mice. **a**. Ovariectomized female BALB/c nude mice were xenografted with Ishikawa^ER+^ cells which were pretreated with **β-**E_2_ or OV-Talin1 or both or neither. And the mice were randomly divided into four groups: OV-NC, OV-Talin1, **β-**E_2_, **β-**E_2_ + OV-Talin1. **b**. Photographs of lesions were taken on the 84th day after inoculating cells into the axilla of mice. **c** and **d**. Lesion growth curves and excised lesion weights were recorded and quantitatively analyzed. **e-g** Protein and mRNA levels of molecular markers related to cell proliferation and angiogenesis in hypodermic endometrial lesions. Data were presented as mean ± SD. ^*^P < 0.05, ^**^P < 0.01, ^***^P < 0.001

To further investigate how Talin1 influenced the growth and survival of ectopic endometrial lesions in vivo, the xenograft mice models were established through subcutaneous inoculation of Ishikawa^ER+^ cells treated with β-E_2_ and/or OV-Talin1 transfection. Since all the model mice had been ovariectomized before injection, the effect of endogenous steroid was avoided. On the 84th day after inoculation, mice were euthanized and the lesion models were completely taken out (Fig. 6a, b).

Compared with the OV-NC group, the final lesion volume in β-E_2_ or OV-Talin1 group was both higher (106.38, 512.63 and 439.33 mm^3^ respectively), while as expected, the co-treatment group of β-E_2_ + OV-Talin1 exhibited the largest lesion with an average of 779.27 mm^3^ (Fig. 6c). Accordingly, β-E_2_ + OV-Talin1 co-treatment group had the highest mean lesion weight (0.634 g), which was, in particular, about twice as heavy as β-E_2_ or OV-Talin1 treated alone (Fig. 6d). Moreover, the lesions were harvested and subjected to western blot detection of markers for cell proliferation and angiogenesis. As presented in Fig. 6e-g, despite a higher expression level was observed in OV-Talin1 or β-E_2_ group than that of OV-NC group, the related molecular markers including PCNA, Ki67, VEGFB and ANGPTL4 were upregulated the most in OV-Talin1 + β-E_2_ group. Collectively, Talin1 and β-E_2_ might synergistically promote the growth and survival of ectopic endometrial lesions through exerting an additive facilitating effect on cell proliferation and neovascularization.

## Discussion

ADS is a commonly encountered estrogen-dependent disorder affecting approximately 20% of women of reproductive age and shows an increased incidence among women with infertility [2, 14, 16]. Despite the recent advantages of diagnostic tools, a shared definition and classification as well as the awareness of the condition are still lacking. Currently, there was no international guideline to follow regarding the completely effective management on ADS, except for hysterectomy [17, 18]. Although the precise etiology and pathogenesis of ADS remain to be further elucidated, several theories or hypotheses addressing the progress of the disease have been put forward [4], including the enhanced invasion and invagination of endometrium into myometrium; metaplasia or differentiation of stem cells; endometrial-myometrial interface disruption (EMID); induction of aberrant local hormones and some genetic or epigenetic modifications.

According to one of the most accepted theories, ADS may result from the invagination of basalis endometrium into the myometrium after crossing an altered or interrupted EMT, a highly specialized hormone-responsive structure, eventually establishing ectopic lesions [19]. Emerging evidence have demonstrated that enhanced endometrial proliferation, more active cell migration and invasion through the EMT phenotype, as well as increased neovascularization were much more common in eutopic and ectopic endometrium of ADS [20–23]. These alterations from endometrial cells have been postulated to be extremely beneficial to endometrium invading into deeper myometrium and maintaining the subsequent growth and survival of adenomyotic ectopic lesions. Notably, during the course of endometrial invagination and implantation, steroid hormones are likely to serve a central role. Particularly, the local supraphysiological estrogen levels may be a preliminary status contributing to the origin of ADS, since it has been manifested that high β-E_2_ initiated and facilitated the microtrauma of EMI as a positive feedback [2, 4]. As described in earlier reports, elevated β-E_2_ induced a shift of epithelial to mesenchymal markers and fostered endometrial cell migration and migration in ADS [8, 24, 25]. Furthermore, our previous study also demonstrated that β-E_2_ could cause hyperproliferation of adenomyotic smooth muscle cells (SMCs) in EMI through activating ER-enhanced RoA-Rock signaling pathway [26, 27]. Also, hyperestrogen has been found to be involved in overexpression of annexin A2 in adenomyotic endometrium, which mediated the angiogenetic process via β-catenin/T-cell factor signaling [28]. Although a growing body of evidence recently linked the pathogenesis of ADS to a remarkable disorder of estrogen metabolism, the molecular mechanisms of this disease still remain largely unelucidated.

In the present study, we further demonstrated that a suitable dose of β-E_2_ exhibited a significant promoting effect on adenomyotic endometrial stromal cell proliferation and pro-angiogenesis. According to reports from Herndon etal. and Guo etal., the molecular mechanism underlying decreased apoptosis and increased proliferation likely derive from excessive E_2_ in adenomyotic endometrium [29, 30]. Huang etal. Also revealed β-E_2_-induced angiogenesis could contribute to ADS by activating the slug/VEGF axis in endometrial epithelial cells [31]. On the premise of not contradicting the previous results, however, our research mainly featured the use of primary isolated and cultured endometrial stromal cells of human ADS instead of merely endometriod adenocarcinoma cell lines, which means better fitting the cellular biological model of ADS. At the same time, we intervened with β-E_2_ in both eutopic and ectopic endometrial stromal cells, which may provide a more powerful supplementary basis for verifying the role of local hyperestrogenism in different positions and stages during the development of ADS. Concomitant treatment with an ER antagonist (Fulvestrant, ICI 182780), which not only abolished the stimulative effects of β-E_2_ on cell proliferation and pro-angiogenesis from the perspective of functional experiment, but also abrogated the expression of markers including PCNA, Ki67, VEGFB and ANGPTL4, further supported an ER-dependent mechanism in ADS. Indeed, these observations may account for elevated β-E_2_-mediated overproliferation and hyperangiogenesis in adenomyotic endometrium. However, whether the key links in β-E_2_-guided ADS can be affected by other factors synchronously, the relevant evidence is still less sufficient.

Talin1, a ubiquitous macromolecular (270-KDa) protein highly enriched at the cell-matrix attachment sites, mostly functions as the key regulator of integrin activation, which is encoded by TLN1 [32]. Since a crucial final step in activating integrin is binding of the N-terminal head domain of Talin1 to the β-integrin cytoplasmic domain, Fadi etal. Recently demonstrated that Talin1-dependent integrin activation could regulate VE-cadherin localization and endothelial cell barrier function, which was critical for vascular sprouting development and stability [33]. Furthermore, an important property of integrin is the modulation of affinity for extracellular ligands, a process termed integrin activation or ″inside-out integrin signaling″. So far, abundant robust evidence has confirmed that Talin1 can bind and activate integrin through modulating its affinity. Once activated, the integrin initiates the activation of FAK, thereby mediating numerous processes concerning cell proliferation, adhesion and mobility [34]. As previous studies implicated, Talin1 was mostly identified to be overexpressed and involved in the progression of multiple human cancers, during which the tumor cell invasion or metastasis was stimulated [35]. On the contrary, Somcyeh etal. Revealed that cytoplasmic expression of Talin1 was associated with advanced pathological features in colorectal cancer, based on the observations that a negative correlation between Talin1 protein level and advanced TNM stage (*P* = 0.028) as well as worse disease specific survival (*P* = 0.011) [36]. As regards the effects of Talin1 in gynecological diseases, it has been reported that Talin1 dysregulation in uterine endometrium of patients with missed abortion would negatively alter the endometrial epithelial cell adhesive capacity during the early stage of pregnancy, thus impeding implantation [37, 38]. Besides, as per available literature, Talin1 was detected to be upregulated in the eutopic and ectopic endometrial glands of ADS by Jiang etal [39],. which was consistent with our previous study. As a partial improvement of Jiang′s research on the specific role and internal mechanism of Talin1, we have previously proved that abnormally overexpressed Talin1 induced EMT in ADS endometrial cells via triggering wnt/β-catenin pathway [11]. Collectively, we have preminarily confirmed that Talin1 could promote the infiltration of adenomyotic endometrium into myometrium. However, whether Talin1 could alter the subsequent proliferation and pro-angiogenesis of endometrial cells acquiring a stromal phenotype to maintain the ectopic implantation and survival after EMT, especially whether Talin1 influences the efficacy of β-E_2_, is still disputed. Therefore, we designed the present study for further investigation.

As expected, our study unveiled that a trend of gradually increasing expression of Talin1 protein from normal uterine endometrium to ADS ectopic endometrium, eutopic endometrium, the corresponding Ctrl_ESC, and primary ADS_Ec_ESC as well as ADS_Eu_ESC cells. More importantly, we provided novel data to present that Talin1 overexpression (OV-Talin1) can serve a positive role in facilitating adenomyotic endometrial stromal cell proliferation and pro-angiogenesis. Histopathologically, ADS is a benign disease, but published work has confirmed that its endometrial cells, especially the stromal cells, are more active in proliferation, migration, invasion and angiogenesis, which are similar to the biological characteristics of tumor cells [4, 19]. Considering the aberrant enrichment of Talin1 has been identified in several tumors and there is strong evidence linking it to oncogenic progress, our findings suggested that Talin1 might also play some distinct roles in the development of ADS. In addition, Pulous etal.demonstrated that Talin1-dependent integrin activation was required for endothelial proliferation and postnatal angiogenesis [33], which may account for the enhanced pro-angiogenesis of endometrial stromal cells transfected with Talin1 overexpression vector in our study. Furthermore, accumulating studies have reported that upregulated Talin1 stimulates overproliferation of glioblastoma multiform cells, ovarian carcinoma cells and HCC cells through triggering FAK signaling [40–42]. Interestingly, results from our previous study indicated that FAK could regulate endometrial stromal cell proliferation, migration and invasion in ADS [43]. Thus, given to the current study, it can be speculated that FAK pathway may also play an important role in Talin1 stimulated proliferation and neovascularization in ADS stromal cells, although more mechanism experiments are needed to conduct.

Based on validating the promoting effects of Talin1 overexpression or β-E_2_ treatment on ADS endometrial stromal cell proliferation and pro-angiogenesis respectively, the synergistic effects of the two were first linked and verified both in vitro and in vivo. Specifically, the ADS_Eu_ESC and ADS_Ec_ESC cells, treated with OV-Talin1 or β-E_2_, acquiring originally higher proliferative and pro-angiogenetic capabilities, presented even more enhanced abilities after co-treated with β-E_2_ plus OV-Talin1. Of note, the xenograft nude mice model was established through inoculation of Ishikawa^ER+^ cells intervened with β-E_2_ or Talin1 overexpression, which further supported the addictive effects of Talin1 on β-E_2_ induced growth and neovascularization of the ectopic endometrial lesions. To our best knowledge, our research has supplemented more data to the factors that alter the efficacy of β-E_2_in the pathogenesis of ADS. Especially for the first time, it has revealed that accompanying up-regulation of Talin1 could positively amply the effects of β-E_2_, and the combination of the two might make it easier for inducing ADS. Our findings may provide a novel therapeutic insight for ADS, for instance, through simultaneous blocking on the effects of β-E_2_ and Talin1.

This study also raised several points that warrant further exploration and improvement. Firstly, concerning the potential molecular mechanism of Talin1 cooperating with β-E_2_, more experimental research is needed. In addition to improving the proliferation and angiogenesis of eutopic and ectopic endometrial stromal cells, it is well worth speculating and verifying whether Talin1 and β-E_2_ serve a joint role during other related pathogenesis of ADS. Furthermore, based on the results from the present study, we are still unable to fully determine the regulatory relationship between Talin1 and β-E_2_. For example, whether β-E_2_ was more likely to be dominant and whether β-E_2_ also regulated the expression and efficacy of Talin1 are under exploration. We should also admit that some figures from the western blot assay were not of very good quality, therefore the densitometric analysis and the final results probably got affected to some extent. In this regard, we need to expand the sample size as much as possible and re-conduct the experiment in our future research. Another limitation in our study is that the model was established through subcutaneous injection of Ishikawa cells, which might fail to completely reshape the clinicopathological process of human ADS, although this method is simpler and has been reported previously.

## Conclusions

Collectively, our study unveiled that β-E_2_ could induce adenomyotic endometrial stromal cell proliferation and pro-angiogenesis. A notable synergistic promoting effect of aberrantly overexpressed Talin1 and β-E_2_ was observed in ADS_Eu_ESC, ADS_Ec_ESC and xenograft models. Therefore, the combined effect of Talin1 and β-E_2_ probably open up a new perspective for elucidating the pathogenesis of ADS and inspiring potential targeted therapeutic strategies.

## Supplementary Information

**Additional file 1: Supplementary Figure S1**. Morphological features of adenomyotic eutopic and ectopic endometrial stromal cells. A.and B. The primary culture of ADS_Eu_ESC and ADS_Ec_ESC cells to the fourth day, respectively. C. The third generation (P_3_) of ADS_Eu_ESC subcultured for 48 h. D. The fifth generation (P_5_) of ADS_Ec_ESC subcultured for 48 h (40x, scale =500 μm).

## Data Availability

The data used to support the findings of this study are available from the corresponding author upon request.

## Abbreviations

ADS: Adenomyosis
ANGPTL4: Angiopoietin-like 4
ADS_Eu_ESC: Adenomyotic eutopic endometrial stromal cells
ADS_Ec_ESC: Adenomyotic ectopic endometrial stromal cells
ADS_Euc: Eutopic endometrium of adenomyosis
ADS_Ec: Ectopic endometrium of adenomyosis
β-E_2_: β-estradiol
CNC: Complete negative control (cells without any treatment)
Ctrl_ESC: Control uterine endometrial stromal cells
Ctrl_En: Control uterine endometrium
EMI: Endometrial-myometrial interface
EMID: Endometrial-myometrial interface disruption
ER^+^: Estrogen receptor positive
EMT: Epithelial-mesenchymal transition
HUVECs: Human umbilical vein endometrial cells
OV-Talin1: Overexpression of Talin1
OV-NC: Overexpression vector for negative control
PCNA: Proliferating cell nuclear antigen
VEGFB: Vascular endothelial growth factor B
GAPDH: Glyceraldehyde-3-phosphate dehydrogenase
