# Supplementary figures and images for: Upregulated Talin1 synergistically boosts β-estradiol-induced proliferation and pro-angiogenesis of eutopic and ectopic endometrial stromal cells in adenomyosis
Source: Reprod Biol Endocrinol. 2021 May 14;19:70. doi: 10.1186/s12958-021-00756-7 (PMC8120781; doi:10.1186/s12958-021-00756-7)

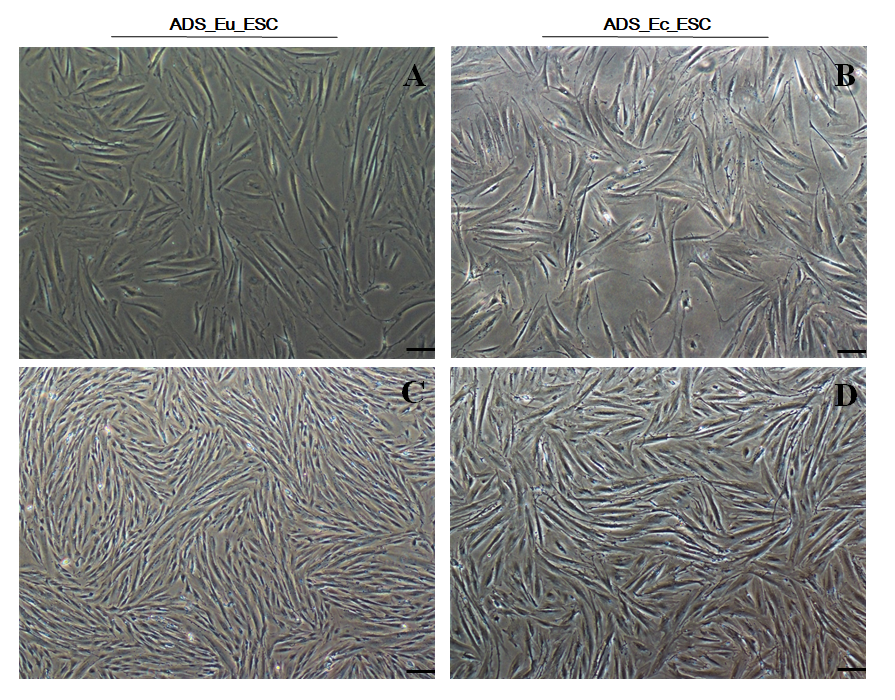

Supplement: Supplementary file 1 — Additional file 1: Supplementary Figure S1. Morphological features of adenomyotic eutopic and ectopic endometrial stromal cells. A.and B. The primary culture of ADS_Eu_ESC and ADS_Ec_ESC cells to the fourth day, respectively. C. The third generation (P3) of ADS_Eu_ESC subcultured for 48 h. D. The fifth generation (P5) of ADS_Ec_ESC subcultured for 48 h (40x, scale =500 μm). [file 12958_2021_756_MOESM1_ESM.png]
